# Functional network activity during errorless and trial‐and‐error color‐name association learning

**DOI:** 10.1002/brb3.1723

**Published:** 2020-06-18

**Authors:** Madoka Yamashita, Tetsuya Shimokawa, Ferdinand Peper, Rumi Tanemura

**Affiliations:** ^1^ Department of Rehabilitation Science Graduate School of Health Sciences Discipline, Life and Medical Sciences Area Kobe University Kobe Japan; ^2^ Center for Information and Neural Networks (CiNet) National Institute of Information and Communications Technology (NICT) Osaka University Suita Japan; ^3^ Rehabilitation Department Asakayama General Hospital Sakai Japan

**Keywords:** cognitive function, fMRI, learning, rehabilitation

## Abstract

**Introduction:**

In cognitive rehabilitation, errorless (EL) and trial‐and‐error (T&E) learning are well‐known methods, but their neural mechanisms are not well known. In this study, we investigated functional magnetic resonance imaging data for healthy adults during EL and T&E learning.

**Methods:**

Participants memorized color‐name associations in both methods using Japanese traditional colors which were unfamiliar to study participants. A functional network analysis was conducted by applying graph theory. We focused on two major cognitive networks: the default mode network (DMN) and the fronto‐parietal network (FPN). Also, we used “within‐network connectivity” and “between‐network connectivity” graph metrics. The former represents the functional connectivity strength of a subnetwork, namely the within‐DMN connectivity and within‐FPN connectivity, while the latter represents the number of links between the DMN and FPN.

**Results:**

The within‐DMN connectivity in T&E learning was significantly higher than in EL learning. The difference between the memory scores of EL and T&E learning weakly correlated with the between‐network connectivity differences between both learning tasks.

**Conclusions:**

Our results suggest that within‐DMN connectivity is important in T&E learning and that the learning benefit differences between EL and T&E approaches potentially relate to the functional integration strength between the DMN and FPN.

## Graphical abstract

Participants memorised colour‐name associations in errorless(EL) and trial‐and‐error(T&E) methods using Japanese traditional colours which were unfamiliar to study participants. We focused on the default mode network (DMN) and the fronto‐parietal network (FPN), and conducted functional network analysis by applying graph theory. Our results suggest that within‐DMN connectivity is important in T&E learning and that the learning benefit differences between EL and T&E approaches possibly relate to the functional integration strength between the DMN and FPN.

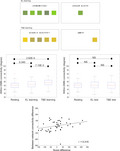

## 1 INTRODUCTION

In cognitive rehabilitation, adapting to the environment by learning (or relearning) skills and knowledge is important for patients with cognitive impairments. Errorless (EL) and trial‐and‐error (T&E) learning are well‐known methods in memory rehabilitation. EL learning aims to prevent mistakes as much as possible; patients are presented with a correct procedure or with correct information before being asked to retrieve target information from long‐term memory to minimize the possibility of erroneous responses (Middleton & Schwartz, 2012; Wilson, 2002). In T&E learning, on the other hand, patients learn by making repeated attempts until achieving success and are instructed to retrieve and process information or episodes during learning (Clare & Jones, 2008; Middleton & Schwartz, 2012). Since Baddeley and Wilson (1994) reported the use of EL learning for amnesic patients and the elderly, it has been used widely in memory rehabilitation; also, its effectiveness for various diseases has been reported (Bertens, Fasotti, Boelen, & Kessels, 2013; Kessels & Hensken, 2009; Tan, Lee, & Lee, 2016). However, some reports describe that the effectiveness of EL learning is knowledge‐specific, while T&E learning has more robust and longer‐term effects than EL learning for young people in their 20s and early‐stage dementia patients (Anderson & Craik, 2006; Dunn & Clare, 2007; Haslam, Gilroy, Black, & Beesley, 2006; Middleton & Schwartz, 2012).

As described above, the effects of EL learning are not consistent among previous studies. The main differences between EL and T&E learning are in the degree of information retrieval and the competition for error elimination; EL learning depends upon these elements much less than does T&E learning.

Additionally, there are differences of opinion currently, as to whether the benefits of EL learning stem from implicit or explicit memory. Implicit memory involves the retrieval of information without conscious recollection, whereas explicit memory involves the conscious recollection of previous experiences (Baddeley & Wilson, 1994; Graf & Schacter, 1985). In other words, implicit memory is a more automatic process than explicit memory, which is a more controlled process that involves episodic memory (Tulving, 2002).

Baddeley and Wilson (1994) suggested that EL learning benefits are based on the relative priority of implicit memory because impaired explicit memory forces amnesic and elderly people to rely more heavily on implicit memory. Some reports also propose that EL learning benefits are due to residual explicit memory (Hunkin, Squires, Parkin, & Tidy, 1998; Tailby & Haslam, 2003); nevertheless, some also believe that both implicit and explicit memory are involved (Page, Wilson, Shiel, Carter, & Norris, 2006).

Although EL and T&E learning are standard methods in cognitive rehabilitation, there has been little agreement on which approach is the most beneficial for specific cognitive dysfunctions because many previous studies are limited to the behavioral level; also, there is a lack of research on the neural mechanisms underlying EL and T&E learning (Clare & Jones, 2008; Mimura & Komatsu, 2010). To the best of our knowledge, only two studies have measured brain activity via functional magnetic resonance imaging (fMRI) focusing on EL and T&E learning (Hammer, Tempelmann, & Munte, 2013; Ueno et al., 2009). Ueno et al. (2009) reported that the precuneus, posterior cingulate cortex, and inferior parietal cortex showed higher activation in the T&E condition than the EL condition using a word stem completion task for patients with diffuse axonal injury and healthy controls, though there was no significant difference between task conditions and between groups. Hammer et al. (2013) reported that the left inferior medial gyrus, bilateral angular gyrus, and superior frontal gyrus showed task condition differences for healthy adults using a face‐name association task. These activated areas in the T&E condition, as reported in previous studies, are parts of the default mode network (DMN) and fronto‐parietal network (FPN); the precuneus, posterior cingulate cortex, inferior parietal cortex, and angular gyrus are members of DMN, and the inferior medial gyrus and superior frontal gyrus are members of the FPN (Cole et al., 2013; Fox & Raichle, 2007; Menon, 2011). However, in both studies, fMRI scans were conducted only during test tasks and only partial brain activity was reported; therefore, the brain‐wide neural mechanisms remain unknown. Hence, investigating the neural mechanisms of information processing in these standard learning methods is important for a more effective and evidence‐based cognitive rehabilitation practice.

Comparisons based on functional networks are helpful for developing learning methods in cognitive rehabilitation. Therefore, in this study, functional network connectivity was examined by applying graph theory to fMRI data to investigate the differences between EL and T&E learning in neural mechanisms on a large‐scale during test and learning tasks. Individuals who learned color‐name associations through EL and T&E learning were evaluated, focusing on two major cognitive networks. The DMN was the first area of focus. On the one hand, the DMN is a task‐negative system that is deactivated, rather than subjected to a resting state, during externally oriented attention tasks and mainly consists of the posterior cingulate cortex, medial prefrontal cortex, medial temporal lobe, and angular gyrus (Fox & Raichle, 2007; Menon, 2011). These DMN brain areas overlap with the activated areas in the T&E condition, as reported in previous studies (Hammer et al., 2013; Ueno et al., 2009).

On the other hand, the DMN is also considered a task‐processing system and is thought to be active during internally oriented mental processes relevant to the theory of mind, self‐evaluation, social cognition, and episodic memory retrieval (Addis, Wong, & Schacter, 2007; Buckner & Carroll, 2007; Chiong et al., 2013; Fox et al., 2005; Menon, 2011; Power et al., 2011; Sporns, 2011a; Spreng, Stevens, Chamberlain, Gilmore, & Schacter, 2010). Moreover, the DMN is thought to act as an episodic buffer that provides a temporary store in which the various components of working memory interact with perceptual information and long‐term memory (Baddeley, 2010; Sestieri, Corbetta, Romani, & Shulman, 2011). We anticipated that the strength of DMN‐associated functional connectivity would be related to the retrieval and processing episodes during T&E learning. Furthermore, we predicted that the strength of DMN‐associated functional connectivity would effectively differentiate between EL and T&E approaches. Investigating the strength of DMN‐associated functional connectivity relating to episodic memory in each learning approach could help to answer questions arising from previous research regarding whether EL learning is based on implicit or explicit memory.

The second area of focus was the FPN. The FPN is a task control system that mainly consists of the lateral prefrontal cortex, posterior parietal cortex, anterior insula cortex, and medial prefrontal cortex (Cole et al., 2013). These areas which belong to the FPN overlap with the activated areas in the T&E condition, as reported in a previous study (Hammer et al., 2013). The FPN is activated during externalized attention tasks and changes the functional connectivity in a variety of brain regions according to the demands of the task to flexibly initiate and adjust cognitive control (Cole et al., 2013; Dosenbach, Fair, Cohen, Schlaggar, & Petersen, 2008; Fox et al., 2005; Power et al., 2011). Consequently, we expected that participants who have greater connectivity between the DMN and FPN in T&E learning than in EL learning would be able to adapt more easily to T&E learning. The present findings are likely to be useful for the analysis of various other diseases, including dementia and schizophrenia because functional network properties in such diseases have been reported in recent years (Menon, 2011).

## 2 MATERIALS AND METHODS

### 2.1 Participants

Forty‐three healthy adults participated in this study (mean age: 34.6, age range: 21–63 years; 19 females, all right‐handers). We confirmed that all participants were not color‐blind to any extent using the Ishihara color blindness test (Ishihara's tests for color blindness; International version, 38 plates edition, Hongo, Handaya, Tokyo) before the experiment, since the experimental tasks require the ability to distinguish colors. All participants provided written informed consent prior to inclusion in the study, according to the tenets of the Declaration of Helsinki, and this study was approved by the institutional review boards of the participating institutions, that is, Kobe University and the Center for Information and Neural Networks (CiNet) of NICT.

### 2.2 Experimental procedures

All participants underwent an fMRI session that included a resting state as the default mode for the first session and four subsequent tasks: EL learning, an EL test, T&E learning, and a T&E test. In the resting state, participants were instructed to rest with their eyes closed for 10 min. The EL or T&E test session was conducted rapidly after the respective learning session. The order of the two learning sessions was randomized for each person.

### 2.3 Administered tasks

Participants were instructed to memorize color‐name associations in learning sessions and to retrieve memories in test sessions. To make the task sufficiently difficult for healthy individuals, Japanese traditional colors were used, which are not simply described as “green” but as “Hiwamoegi,” for example. There are hundreds of Japanese traditional colors that are unfamiliar to most Japanese people, including the participants in our study, as these colors are only commonly known by experts in the kimono industry. Previous studies concerning EL and T&E learning typically used word‐stem completion tasks that mainly involved verbal information. In this study, tasks were used that involved verbal and visual information to activate a larger part of the brain, rather than a specific brain region, so that the test resembles a more realistic situation.

Participants memorized one target color among five candidate colors presented as rectangles and labeled by the target color name; in total, they memorized 10 color‐name associations for EL and T&E approaches. The target colors in each learning session were different (Yamashita, Shimokawa, Peper, Uchida, & Tanemura, 2015).

The hue, saturation, and lightness (HSL) criteria were used to select a target color; each value was rescaled to be between 0 and 255 in accordance with PowerPoint 2013 (Microsoft Corporation) software conventions. Target colors were selected randomly from Japanese traditional colors; color choices were restricted to those with lightness values of 100–200 to avoid making the discrimination of a target color from the other four candidate colors too difficult. Hues were selected such that the whole color palette was represented evenly in the EL and T&E learning sessions. Five candidate colors with the same hue and lightness values, but different values of saturation, were generated.

For each target color, the set of five candidate colors was generated, including the target color, in the following way. First, under the assumption that the target color saturation value is S, a set of all saturation values S − 35n and S + 35m was generated, with n and m being integers chosen such that all saturation values were between 0 and 255. The amount of saturation change was assigned an arbitrary value of 35 to make the discrimination between the five candidate colors moderately difficult. It can easily be verified that this set contains seven or eight components. For example, when the saturation value of the target color is S = 46, then the seven saturation values are 11, 46, 81, 116, 151, 186, and 221. Second, all sets of five consecutive saturation values were selected from the set that contained S. Consequently, in the previous example, there would be 2 sets, {11, 46, 81, 116, 151} and {46, 81, 116, 151, 186}, because these would be the only sets containing 46. Third, one of the sets generated in the previous step was randomly selected. In the previous example, the set {11, 46, 81, 116, 151} would be selected. The target value 46 in this set would be second from the left. By listing all possibilities, it can be demonstrated that the position of the target value in a set is uniformly distributed, provided the target values of the saturation over all colors are uniformly distributed. Hence, there was no bias regarding the position of the target color.

All accompanying words presented on the screen were in Japanese and the color names were presented in a phonetic (Japanese) alphabet, that is, not using Chinese characters, to avoid associations with particular meanings. The five candidate colors were presented by rectangles and arranged side by side on the screen, and the words were presented above the colors. On each trial, the five candidate colors’ locations were set randomly so that target colors were presented in equal probability across the five locations.

### 2.4 Errorless learning

**FIGURE 1:**
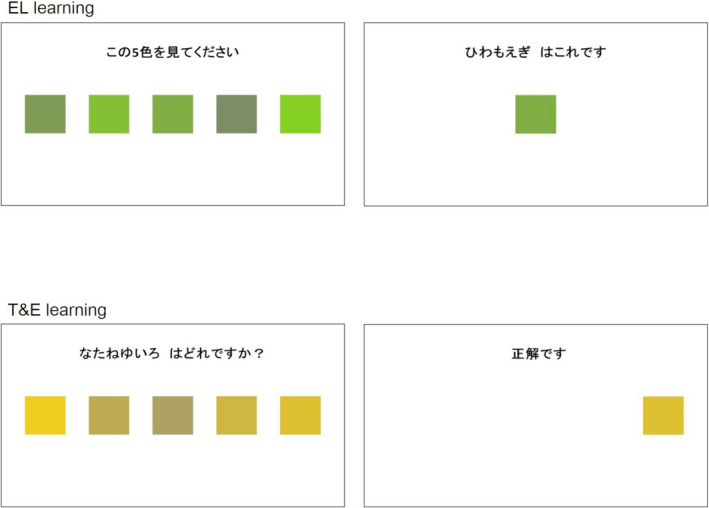
Examples of pictures presented in EL learning (above) and T&E learning (below) are shown. In EL learning, the Japanese sentence on the left means “Watch these 5 colors, please” and that on the right means “Hiwamoegi is this color.” In T&E learning, the Japanese sentence on the left means “Which color is Nataneyu‐iro?” and that on the right means “Correct.” Abbreviation: EL, errorless; T&E, trial‐and‐error

Participants were presented with five candidate colors that included the target color as well as the sentence “Watch these 5 colors, please” for 9 s. Subsequently, only the target color remaining in the same location as on the prior screen accompanied by that color name was presented for 6 s; for example, the phrase “Hiwamoegi is this color” was used as shown in Figure 1 (above). For all 10 color‐name associations, the same procedures were followed. These procedures for the 10 color‐name associations were repeated four times randomly (randomizing the order of the 10 target colors and the locations of the five rectangles). Thus, in EL learning, participants learned by viewing the presented screen.

### 2.5 Trial‐and‐error learning

Participants were presented with five candidate colors that included the target color as well as a question such as “Which color is Nataneyu‐iro?” They selected the target color by pushing buttons corresponding to the locations of the five candidate colors. Until participants answered correctly, the same screen continued to be presented. Due to the repetitive nature of this process, the time for each T&E learning session differed for each person. When participants answered correctly, only the target color in the same location as on the prior screen as well as the word “correct” was presented on the next screen for 6 s (Figure 1, below). The same procedures were performed for the 10 color‐name associations as in EL learning. Additionally, these procedures for the 10 color‐name associations were repeated four times randomly (randomizing the order of the 10 target colors and the locations of the five rectangles). Thus, in T&E learning, participants learned through an active trial‐and‐error approach that involved pushing buttons.

### 2.6 Errorless and trial‐and‐error tests

**FIGURE 2:**
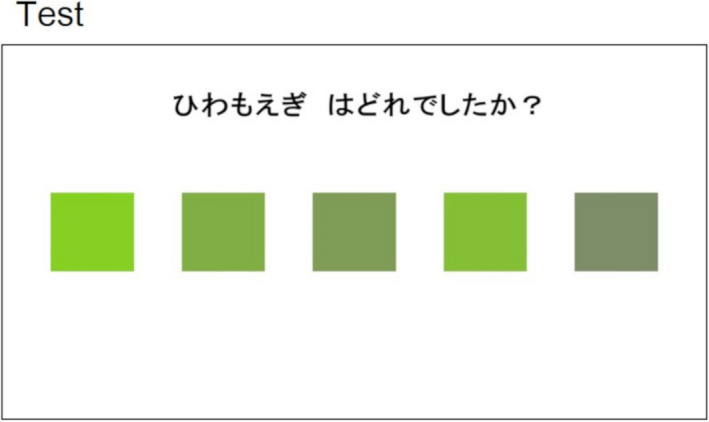
An example of a picture presented in test sessions is shown. The Japanese sentence means “Which color is Hiwamoegi?”

Participants were presented five candidate colors for 9 s that were the same as in the learning sessions accompanied by a question such as “Which color is Hiwamoegi?” Participants selected the target color by pushing buttons corresponding to the locations of the five candidate colors while the question was presented (Figure 2). In the test session, the results of whether each answer was correct or incorrect were not presented to avoid learning in the test session. Similar to the learning session, questions for a total of 10 color‐name associations were repeated four times randomly (randomizing the order of the 10 target colors and the locations of the five rectangles). The EL score was calculated by multiplying the number of total correct answers in the EL test sessions by 10. The same approach was used for the T&E score calculation.

### 2.7 Functional magnetic resonance imaging data acquisition

Whole‐brain fMRI images were acquired by a 3T fMRI scanner Magnetom Trio (Siemens AG). For functional images, a gradient‐echo echo‐planer imaging sequence was used, represented by 3.0‐mm‐thick axial slices consisting of 45 contiguous slices obtained every 3 mm with the following parameters: repetition time = 3,000 ms, echo time = 30 ms, flip angle = 80°, field of view = 192 mm × 192 mm, and pixel matrix = 64 × 64 with 3 × 3 × 3 mm voxels. Stimuli were presented and synchronized with the scanner sequence using Presentation software (Neurobehavioral Systems, San Francisco, California, USA, http://www.neurobs.com/, RRID:SCR_002521). Participants in the scanner viewed stimuli on a projected screen via a mirror.

### 2.8 Network analysis

#### 2.8.1 Preprocessing

Image preprocessing was carried out using SPM8 (Wellcome Department of Cognitive Neurology, London, UK, http://www.fil.ion.ucl.ac.uk/spm/, RRID:SCR_007037) on MATLAB‐R2014a (MathWorks, Sherborn, Massachusetts, USA, http://www.mathworks.com/products/matlab/, RRID:SCR_001622). The first five images in the scan sequence were excluded from the analysis to rule out the nonequilibrium effects of magnetization; the remaining functional images were realigned to correct for head movement. After realignment (motion correction), functional images were slice‐timing corrected and normalized using the Montreal Neurological Institute (MNI) template (http://imaging.mrc‐cbu.cam.ac.uk/imaging/MniTalairach/, RRID:SCR_001965). Smoothing was not conducted to avoid spurious correlations between adjacent nodes (Alakörkkö, Saarimäki, Glerean, Saramäki, & Korhonen, 2017). Additionally, functional images were high‐pass‐filtered (0.01Hz) to remove slow frequency drifts from the fMRI signal.

#### 2.8.2 Graph properties

In this study, a graph‐theoretical analysis was conducted using MATLAB‐R2014a. Functional network graphs were constructed whereby nodes represented brain regions and undirected links represented connections between brain regions. Also, 264 functional nodes were used as the regions of interest (ROIs), each of which was a sphere with a 10 mm diameter that was centered on the coordinates defined by Power et al. (2011). The nodes were assigned to the 10 functional subnetworks defined by Cole et al. (2013); of these 10, the DMN and FPN subnetworks were used for network analysis.

The time‐series ensemble average was calculated for the individual voxels with 3 mm sides in each ROI; this was considered the representative time series of the ROI. Based on the 264 representative time series, pairwise correlation coeffcients were calculated and organized as a correlation matrix (264 × 264 square matrix); then, an adjacency matrix (264 × 264 square matrix) was calculated based on the correlation matrix for each of the participant tasks. If an element of the correlation matrix was larger (or smaller) than a threshold, then the corresponding element of the adjacency matrix was set to 1 (or 0), meaning that there was a link (or no link) between the corresponding nodes in the graph (Yamashita et al., 2015). Given that our goal was to compare experimental participants with each other, the threshold was set individually for each person such that the top 25% of the correlation values were represented as links in the graph (Fornito, Zalesky, & Bullmore, 2016; Rubinov & Sporns, 2010; Sporns, 2011b). Thus, the brain networks of all participants had an equal number of links, though the distributions of these links differed.

To evaluate the relations between different parts of the graph, the degree of the node was used as a principal graph property; the degree of a node is the number of links that connect the node to the rest of the graph. The within‐network connectivity is then defined as the average of total links across all nodes within the corresponding subnetwork, whereby all links adjacent to a node within the subnetwork are counted and averaged per node; this incorporates the links connecting to nodes inside and outside the subnetwork. A large within‐network connectivity is thought to reflect enhanced activity in the corresponding subnetwork (Sporns, 2011b). The between‐network connectivity is defined as the number of links between these two subgraphs; when applied to brain networks, it gives an indication of the degree of information flow between the subnetworks in the corresponding brain areas.

Two graph metrics were analyzed: the within‐network connectivity of the DMN and FPN, called the within‐DMN connectivity and within‐FPN connectivity, respectively, and the between‐network connectivity between the DMN and FPN. The DMN contained 58 nodes, whereas the FPN contained 25 nodes.

### 2.9 Statistical analysis

Statistical analyses were performed using MATLAB‐R2014a, whereby significance was determined by a threshold of *p* < .05. The differences between EL and T&E scores were analyzed using the Wilcoxon signed‐rank test. The differences in the within‐DMN (FPN) connectivity and between‐network connectivity between the EL and T&E states were assessed using the Friedman test, and a Bonferroni correction was applied to correct for multiple comparisons. The same approach was used to compare both EL and T&E to their respective resting states. The correlations between cognitive performance and the graph metrics were analyzed using the Spearman correlation coefficient.

## 3 RESULTS

### 3.1 Behavioral analysis

**FIGURE 3:**
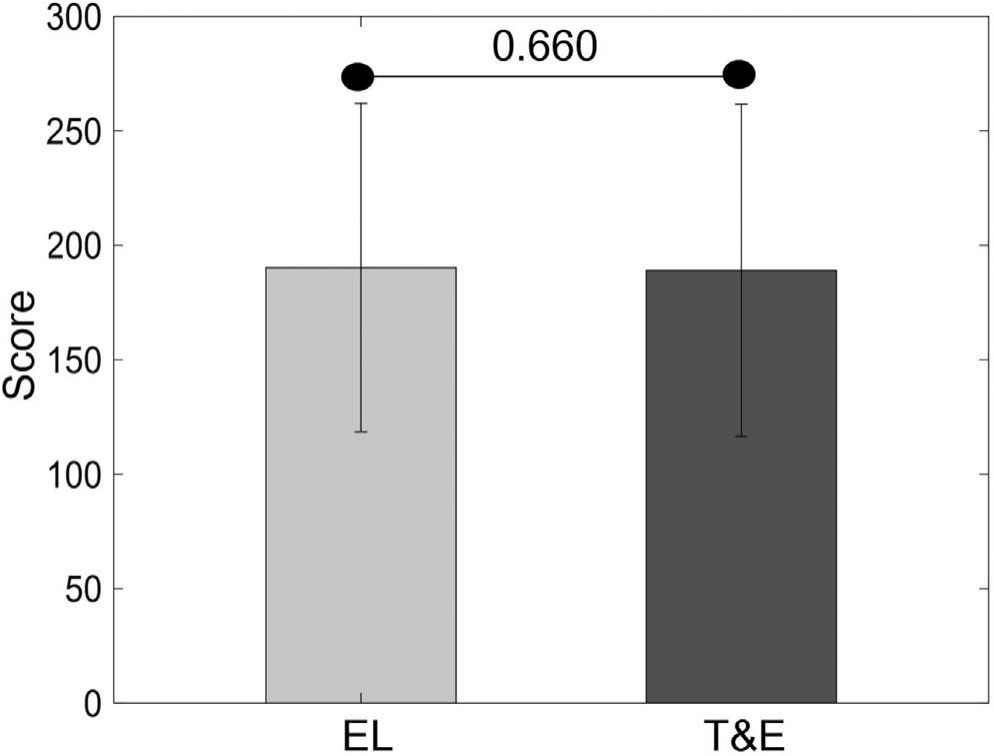
The mean (±*SD*) EL and T&E scores of all participants are shown. The black horizontal line and the associated number represent the *p*‐value of the Wilcoxon signed‐rank test. There was no significant difference between EL and T&E scores. EL, errorless; *SD*, standard deviation; T&E, trial‐and‐error

The mean (±standard deviation) scores of all participants in the EL (190 ± 72) and T&E (189 ± 73) test sessions are shown in Figure 3. There were no significant differences between the EL and T&E scores [*Z* = −0.440, *p* = .660].

### 3.2 Within‐DMN connectivity

#### 3.2.1 Learning tasks

**FIGURE 4:**
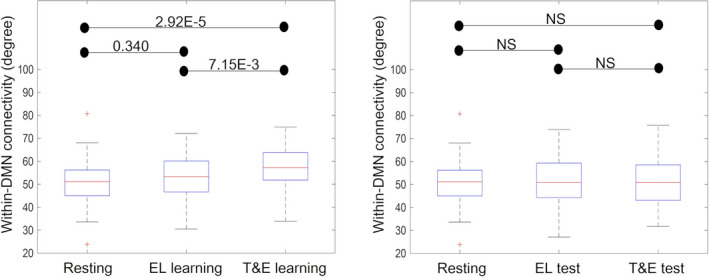
The within‐DMN connectivity in resting, EL learning, and T&E learning states (left) and the within‐DMN connectivity in resting, EL test, and T&E test states (right) are shown. The boxplots show the median, interquartile range, and range of the within‐DMN connectivity in each state. Each black horizontal line and the associated number represent the *p*‐value of the Friedman test after applying a Bonferroni correction for multiple comparisons. Each black horizontal line and the associated “NS” indicate a statistically nonsignificant difference. The red + signs in the resting state data indicate outliers. The within‐DMN connectivity in T&E learning was significantly higher than that in resting and EL learning states. There were no significant differences between the within‐DMN connectivity in EL learning and resting states. There were no significant differences among the within‐DMN connectivity in resting, EL test, and T&E test states. DMN, default mode network; EL, errorless; T&E, trial‐and‐error

There were significant differences among the within‐DMN connectivity in resting, EL learning, and T&E learning states [*Z* = 20.4, *p* = 3.68E‐5]. The multiple comparison results showed that the within‐DMN connectivity in T&E learning (57.5 ± 9.6) was significantly higher than that at rest (50.9 ± 10.3) and that in EL learning (53.9 ± 9.0) [*Z* = −0.954, *p* = 2.92E‐5; *Z* = −0.651, *p* = 7.15E‐3, respectively]. There was no significant difference between the within‐DMN connectivity in EL learning and that at rest [*Z* = −0.302, *p* = .340] (Figure 4, left).

#### 3.2.2 Test tasks

There were no significant differences among the within‐DMN connectivity in resting (50.9 ± 10.3), EL test (52.2 ± 10.5), and T&E test states (50.7 ± 10.4) [*Z* = 1.44, *p* = .486] (Figure 4, right).

### 3.3 Within‐FPN connectivity

#### 3.3.1 Learning tasks

**FIGURE 5:**
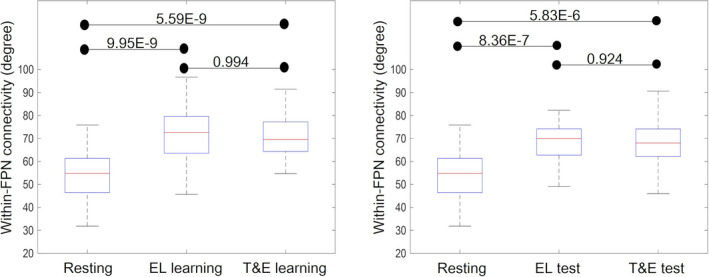
The within‐FPN connectivity in resting, EL learning, and T&E learning states (left) and the within‐FPN connectivity in resting, EL test, and T&E test states (right) are shown. The boxplots show the median, interquartile range, and range for the within‐FPN connectivity in each state. Each black horizontal line and the associated number represent the *p*‐value of the Friedman test after applying a Bonferroni correction for multiple comparisons. The within‐FPN connectivities in EL and T&E learning were significantly higher than those in resting states. There were no significant differences between the within‐FPN connectivity in EL and T&E learning. The within‐FPN connectivities in EL and T&E test states were significantly higher than those in resting states. There were no significant differences between the within‐FPN connectivity in EL and T&E test states. EL, errorless; FPN, fronto‐parietal network; T&E, trial‐and‐error

There were significant differences among the within‐FPN connectivity in resting, EL learning, and T&E learning states [*Z* = 47.8, *p* = 4.24E‐11]. The multiple comparison results revealed that the within‐FPN connectivity in EL learning (71.5 ± 11.6) and in T&E learning (71.2 ± 9.2) was significantly higher than that in a resting state (54.1 ± 11.1); [*Z* = −1.28, *p* = 9.95E‐9; *Z* = −1.30, *p* = 5.59E‐9, respectively]. There were no significant differences between the within‐FPN connectivity in EL and T&E learning [*Z* = −2.33E‐2, *p* = .994] (Figure 5, left).

#### 3.3.2 Test tasks

There were significant differences among the within‐FPN connectivity in resting, EL test, and T&E test states [*Z* = 32.8, *p* = 7.61E‐8]. The multiple comparison results indicated that the within‐FPN connectivity in EL test (68.4 ± 8.2) and in T&E test (68.2 ± 10.2) states was significantly higher than that in resting states (54.1 ± 11.1); [*Z* = −1.10, *p* = 8.36E‐7] and [*Z* = −1.02, *p* = 5.83E‐6], respectively. There were no significant differences between the within‐FPN connectivity in EL test and T&E test states [*Z* = 8.14E‐2, *p* = .924] (Figure 5, right).

### 3.4 Between‐network connectivity

#### 3.4.1 Learning tasks

**FIGURE 6:**
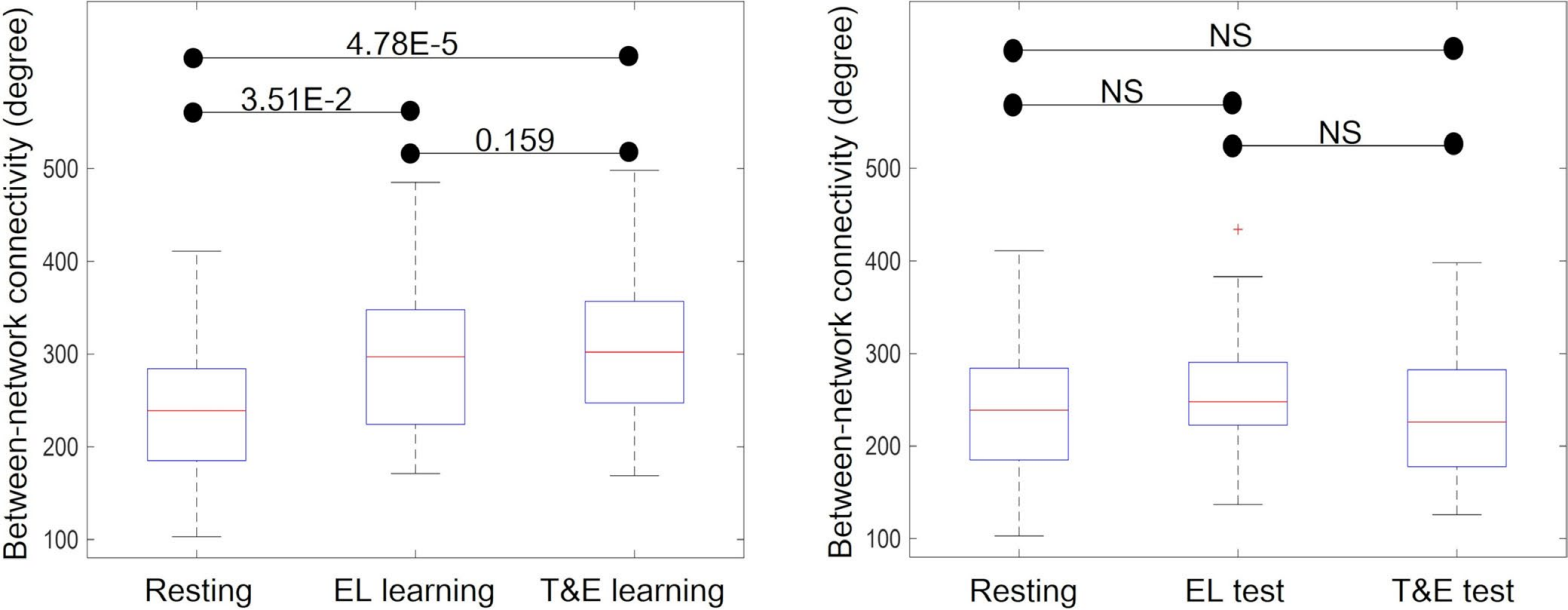
The between‐network connectivity in resting, EL learning, and T&E learning states (left) and the between‐network connectivity in resting, EL test, and T&E test states (right) are shown. The value on the *y*‐axis shows an indication of the degree of information flow between the DMN and FPN. The boxplots show the median, interquartile range, and range for the internetwork degree in each state. Each black horizontal line and the associated number represent the *p*‐value of the Friedman test after applying a Bonferroni correction for multiple comparisons. Each black horizontal line and the associated “NS” indicate a statistically nonsignificant difference. The red + sign in the EL state data indicates an outlier. The between‐network connectivities in EL and T&E learning were significantly higher than those in resting states. There were no significant differences between the between‐network connectivity in EL and T&E learning. There were no significant differences among the between‐network connectivity in resting, EL test, and T&E test states. DMN, default mode network; EL, errorless; FPN, fronto‐parietal network; T&E, trial‐and‐error

There were significant differences among the between‐network connectivity in resting, EL learning, and T&E learning states [*Z* = 18.7, *p* = 8.51E‐5]. The multiple comparison results revealed that the between‐network connectivity in EL learning (287.8 ± 77.0) and T&E learning (305.8 ± 77.1) states was significantly higher than that in a resting state (238.2 ± 71.9); [*Z* = −0.535, *p* = 3.51E‐2] and [*Z* = −0.930, *p* = 4.78E‐5], respectively. There were no significant differences between the between‐network connectivity in EL learning and T&E learning states [*Z* = −0.395, *p* = .159] (Figure 6, left).

#### 3.4.2 Test tasks

There were no significant differences among the between‐network connectivity in resting (238.2 ± 71.9), EL test (259.7 ± 63.0), and T&E test states (237.9 ± 68.2) states [*Z* = 2.93, *p* = .231] (Figure 6, right).

### 3.5 Relationship to cognitive performance

In EL learning, the within‐FPN connectivity was significantly higher than that in the resting state, but there was no significant difference in the within‐DMN connectivity. However, in T&E learning, both the within‐DMN and within‐FPN connectivity were significantly higher than those in resting states. Further, the within‐DMN connectivity in T&E learning was higher than that in EL learning, but there was no significant difference in within‐FPN connectivity.

**FIGURE 7:**
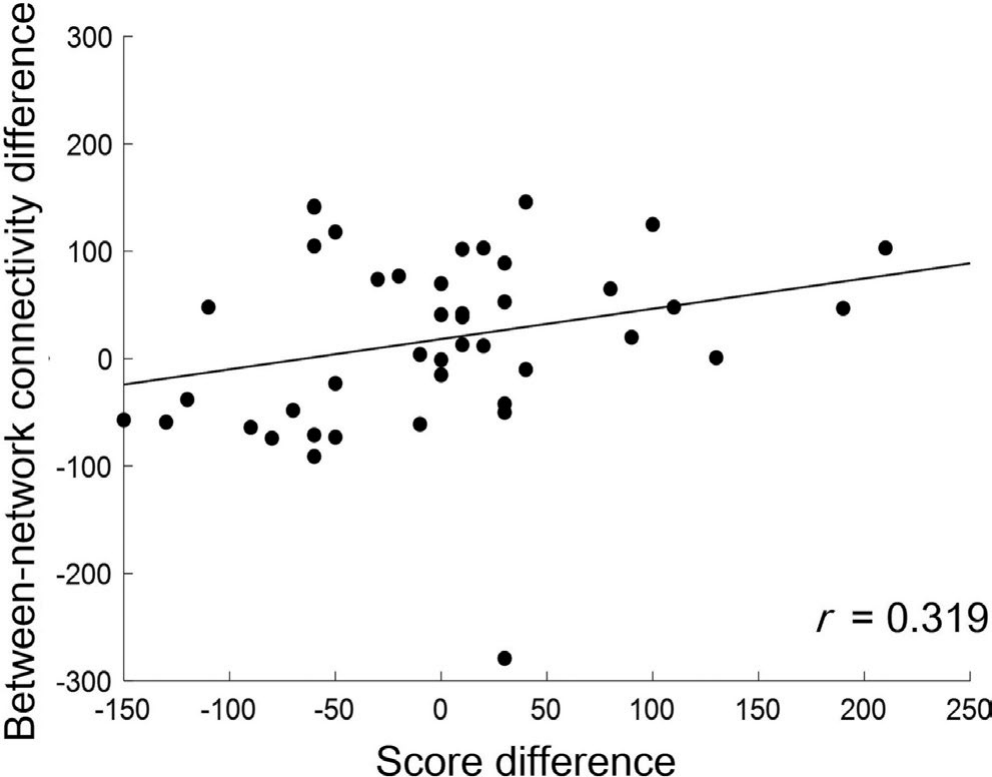
The between‐network connectivity differences between EL and T&E learning, as a function of score differences between EL and T&E methods, are shown. The dots correspond to the 43 participants. The correlation between the between‐network connectivity differences and score differences were analyzed using the Spearman correlation coefficient. The *r*‐value indicates the correlation coefficient. The between‐network connectivity differences showed a significant correlation with the score differences. EL, errorless; T&E, trial‐and‐error. *p* = .037

The possibility that the between‐network connectivity differences between EL and T&E learning are related to the learning benefit differences between them was investigated. The differences in learning benefits were determined by subtracting the EL score from the T&E score (herein termed the score difference). The between‐network connectivity differences were calculated by subtracting the between‐network connectivity in EL learning from that in T&E learning. The between‐network connectivity differences were weakly correlated with score differences [*r* = .319, *p* = .037] (Figure 7). Table 1 summarizes these results.

**TABLE 1:** A comparison of within‐network connectivity and between‐network connectivity for EL, T&E, and resting states in both learning and test tasks

	Within‐network connectivity		Between‐network connectivity
	DMN	FPN	
Learning	EL ~ rest	EL > rest	EL > rest
	T&E > rest	T&E > rest	T&E > rest
	T&E > EL	T&E ~ EL	T&E ~ EL
Test	EL ~ rest	EL > rest	EL ~ rest
	T&E ~ rest	T&E > rest	T&E ~ rest
	T&E ~ EL	T&E ~ EL	T&E ~ EL

Note

The expression “A > B” indicates that task A corresponds to a higher degree than task B. A tilde mark indicates that no significant differences were observed.

Abbreviations: DMN, default mode network; EL, errorless; FPN, fronto‐parietal network; T&E, trial‐and‐error.

## 4 DISCUSSION

In this report, the neural mechanisms during learning and testing in EL and T&E states were investigated, as well as their relationship with cognitive performance. A functional network analysis was performed via graph theory focusing on the DMN and the FPN. The graph property used for the network analysis was based on the number of links between nodes or subnetworks, which represents the functional connectivity strength.

The main findings of the present study are summarized as follows: (a) the within‐DMN connectivity in T&E learning was significantly higher than that in the resting state and EL learning, (b) the within‐FPN connectivities in all four tasks (learning and testing for both EL and T&E approaches) were significantly higher than those in resting states, and (c) the between‐network connectivity differences between EL and T&E learning showed weak correlations with EL and T&E learning score differences.

The within‐DMN connectivity corresponding to T&E learning was significantly higher than those in resting states and EL learning. This result indicates that within‐DMN connectivity is strengthened in T&E learning and can differentiate between EL and T&E learning, thus supporting our hypothesis. The DMN has been reported to act during episodic memory retrieval and serve as an episodic buffer (Addis et al., 2007; Buckner & Carroll, 2007; Sestieri et al., 2011; Sporns, 2011a). The main differences between EL and T&E learning are in the degree of episodic memory retrieval during learning and manipulating across information for error elimination; T&E learning needs these elements much more than EL learning. These results suggest that strengthened DMN‐associated information flow in T&E learning contributes to error elimination via episodic memory retrieval and manipulation.

In feedback learning theory, participants learn stimulus–response associations through the feedback corresponding to their responses in every trial (Cohen, Wilmes, & Vijver, 2011). During feedback learning, participants correct their subsequent responses momentarily according to preceding error‐related feedback. Participants need to correct their erroneous responses to achieve correct answers in both feedback learning and T&E learning. However, compared to feedback learning, T&E learning requires that people more so use endogenous information and process competing information explicitly. This is because individuals need to continue to hold their own responses and the respective feedback in working memory and integrate the information across several trials for error elimination until they reach the correct answer. T&E learning is distinct from feedback learning in terms of the degree of conflict and working memory load for error elimination. However, activity change due to error‐related feedback is reportedly generated by the medial prefrontal and posterior cingulate cortices which belong to the DMN and are associated with performance monitoring and adjustment (Cohen & Ranganath, 2007; Cohen et al., 2011; Müller, Möller, Rodriguez‐Fornells, & Münte, 2005; Nieuwenhuis, Slagter, von Geusau, Heslenfeld, & Holroyd, 2005).

The results of these studies, as well as the strengthened DMN‐associated information flow in T&E learning (as found in the present study), suggest that DMN‐associated areas are involved in performance monitoring and processing erroneous responses.

In the test tasks, there were no significant differences among the within‐DMN connectivity in resting, EL, and T&E states. In the present study, the EL and T&E test protocols were the same in the sense that participants were instructed to only select the color corresponding to a presented color‐name from candidate colors that were the same as the learning phase sets, without being notified whether their selections were correct or incorrect. Participants required less retrieval and manipulation of information for performance monitoring and error elimination because no feedback and T&E were involved in both EL and T&E test tasks.

In the two previous studies investigating brain activity during EL and T&E test tasks using fMRI, the difficulty between EL and T&E test tasks was modified to make participants select targets from more error‐induced candidates as distracters in the T&E test compared to the EL test. One study reported higher activation in the precuneus and the posterior cingulate cortex in the T&E test than in the EL test for healthy controls, but the differences were not significant (Ueno et al., 2009). The authors associated the higher activation in the T&E test with memory retrieval. The other study reported that the left inferior medial gyrus, bilateral angular gyrus, and superior frontal gyrus showed significantly higher activation in the T&E test than in the EL test for healthy adults (Hammer et al., 2013). The authors associated this higher activation with executive control of memory processing.

These activated areas in the above studies are in regions involved in the DMN (Fox & Raichle, 2007; Menon, 2011), which would seem to indicate that brain areas in the DMN play a role in memory retrieval and processing for error elimination. Although we cannot make a direct comparison because these previous results were derived from activity level analysis while the present results were derived from correlations of activity time series, both demonstrate the relationship between DMN regions and error elimination in the T&E condition. The possibility remains that the strength of within‐DMN connectivity could reveal the difference between EL and T&E test tasks if each test task is set with different difficulties of error elimination.

In contrast, the learning and test tasks in the T&E condition were similar in that both involved selecting from the same candidate colors, except in the learning phase the selection was repeated until a correct answer was obtained. This similar protocol between the learning and test tasks in the T&E condition may have led to the practice effect, which could have caused the lack of strengthened within‐DMN connectivity in the T&E test tasks and no significant difference between the EL and T&E test tasks.

Furthermore, the lack of strengthened within‐DMN connectivity in both the EL and T&E test tasks compared to resting state indicates that the strengthened within‐DMN connectivity in T&E learning did not reflect the action of pushing buttons because participants pushed buttons in both EL and T&E test tasks. Overall, we believe that the importance of the DMN for error elimination increases according to the increasing need for retrieving and processing memory in the present task.

The within‐FPN connectivities in all four tasks were significantly higher than that in the resting state, but the within‐FPN connectivity showed no significant differences between the EL and T&E states in either the learning or test tasks. This indicates that strengthened within‐FPN connectivity is common to all four task states. Notably, the FPN has been termed as a task‐positive network and FPN nodes have stronger functional connectivity to each other during attention‐demanding cognitive tasks compared to resting state (Fox et al., 2005; Power et al., 2011). In the present study, participants were presented with stimuli in all four tasks, excluding the resting state, and were required to maintain and integrate the presented stimuli by focusing their attention on them. Therefore, the strength of within‐FPN connectivity did not differ between EL and T&E states. Furthermore, we suggest that the strength of within‐FPN connectivity cannot be influenced by whether stimuli are processed passively or actively during an externalized attention task. Given that the FPN includes a flexible hub region and can change its functional connectivity with a variety of brain regions according to task context, it seems important to investigate with which functional networks the FPN changes its functional connectivity (Cole et al., 2013).

Based on the results regarding within‐network connectivity here, strengthening within‐DMN connectivity tends to occur more in T&E learning, while strengthening within‐FPN connectivity occurs in both EL and T&E learning.

As mentioned in the introduction, EL learning aims to prevent mistakes (Wilson, 2002). Clare and Jones (2008) demonstrated that EL learning is not genuinely errorless but rather error‐reducing. Based on this theory, it can be reasoned that EL learning and T&E learning are not classified clearly and that the EL method implies a relative EL state whereas T&E alludes to a relatively error‐filled state. The present results suggest that if learning protocols become more T&E based, the importance of the DMN in T&E learning becomes higher, and vice versa.

Previous studies have suggested various philosophies on whether the processes of EL learning are based on implicit or explicit memory. Though the DMN has been reported to be related to explicit memory (Addis et al., 2007; Buckner & Carroll, 2007; Sestieri et al., 2011; Sporns, 2011a), the present results show that DMN‐associated information flow is less important in EL learning than in T&E learning. These results indicate that EL learning is based on explicit memory to a lesser extent than T&E learning. Thus, if learning tasks take an EL approach, the learning processes would be, to a lesser extent, based on explicit memory compared to T&E learning.

The results from the present study also show that the degree of functional connectivity between the DMN and FPN is not significantly different between EL and T&E states in either the learning or test tasks and does not differentiate between EL and T&E states. However, there was a weak relationship between cognitive performance and the degree of functional connectivity between the DMN and FPN; more specifically, the between‐network connectivity differences between EL and T&E learning showed weak correlation with score differences between the EL and T&E. This finding indicates that participants who have a stronger functional connectivity between the DMN and FPN in T&E learning than in EL learning could receive more benefits from T&E learning than EL learning.

Our results in the learning tasks showed the importance of the DMN in T&E learning as described above, and the FPN reportedly maintains task‐relevant information and changes the functional connectivity in a variety of brain regions according to the task contexts to flexibly initiate and adjust cognitive control (Cole et al., 2013; Dosenbach et al., 2008; Fox et al., 2005; Power et al., 2011). It is thought that stronger DMN and FPN correlation offers a flexible adaptation to T&E learning, which requires actively maintaining, retrieving, and processing information to eliminate errors. Increased internetwork integration is reported to be necessary for successful working memory performance with greater cognitive effort (Cohen & D’Esposito, 2016; Kitzbichler, Henson, Smith, Nathan, & Bullmore, 2011). Additionally, co‐activation between the DMN and FPN plays a role in goal‐directed and introspective cognitive control related to episodic memory (Spreng et al., 2010).

Hammer et al. (2013) reported that the fronto‐temporal‐parietal network (which they inferred from activated areas in their experiment) was activated during correct‐responding test tasks under the T&E condition with more error‐induced distracters. The fronto‐temporal‐parietal network mentioned in their report involves brain regions of the DMN and FPN: the anterior cingulate cortex, posterior cingulate cortex, bilateral medial temporal sulcus, and bilateral angular gyrus. Though they analyzed test phase scanning data, their results could support our notion that functional correlation between the DMN and FPN supports better performance under error‐inducing conditions.

Furthermore, Bjork (1994) along with Bjork and Bjork (2011) suggests that making the learning method challenging by involving explicit information retrieval and interpretation leads to a stronger learning effect. The T&E approach requires active effort to retrieve and process information during learning, whereas in the EL approach, participants receive correct answers passively (Clare & Jones, 2008; Middleton & Schwartz, 2012; Page et al., 2006). Based on the above description, it can be reasoned that the more participants can flexibly adapt to T&E learning by strengthening the functional connectivity between the DMN and FPN, the more they can learn via T&E learning, compared to EL learning.

For the above reasons, we suggest that integration between the DMN and FPN could strengthen the effect of T&E learning. However, given the small sample size for a correlation analysis, our results for the correlation between score differences and the between‐network connectivity differences should be considered tentative.

Though both the DMN and FPN are necessary for T&E learning, the DMN is less essential for EL learning, according to the present results. Notably, DMN dysfunction is reported in patients with neuropsychological disorders such as dementia and schizophrenia (Menon, 2011; Zhang & Raichle, 2010). Hence, it can be argued that patients with DMN dysfunction are able to learn more successfully in a more EL state; further, both recovery from DMN dysfunction and the functional integration of the DMN and FPN could be trained by gradually making learning protocols more T&E‐based. However, it is difficult to say which learning method is more effective. Overall, it is important to adjust the learning task to the needs of individuals because the important brain functional networks involved change according to the learning method.

## 5 CONCLUSIONS

This investigation into the neural mechanisms underlying EL and T&E learning, accomplished by analyzing functional brain networks using fMRI, shows that within‐DMN connectivity differentiates between EL and T&E learning and is of more importance in T&E learning. Further, the differences in learning benefits between EL and T&E learning for healthy adults possibly relate to the functional integration strength between the DMN and FPN. Importantly, a relationship was revealed between functional network connectivity and cognitive performances in the field of rehabilitation. Hence, the present results may resolve some questions arising from previous research on EL and T&E learning; furthermore, they may be used to tailor individual learning methods on a case by case basis.

The current study has several limitations. First, we only performed a standard realignment for head motion correction in the fMRI data preprocessing, which could have led to spurious patterns in correlation. Additionally, given the small sample size for a correlation analysis, our results for the correlation between connectivity metrics and behavioral performance should be treated with caution. As mentioned above, EL learning and T&E learning are not classified clearly; they are relative relationships. Thus, the lack of clear definitions for EL and T&E learning is also limitations of the present study. If the definitions were to be revised in the future, this analysis would need to be modified to reflect those changes, and further research will be needed to investigate how the functional connectivity of the DMN or between the DMN and FPN change upon regulating the degree of EL or T&E. Moreover, examining the functional connectivity of additional networks is strongly encouraged to obtain further insight into efficient learning approaches. Overall, though the findings in this study are based on healthy adults, they should prove useful for the rehabilitation of elderly people and patients with neuropsychological disorders as well.

## CONFLICT OF INTEREST

The authors report no conflict of interest.

## AUTHOR CONTRIBUTIONS

MY, TS, and RT developed the concept and design of the study. MY and TS contributed to the acquisition, analysis, and interpretation of data. MY, TS, FP, and RT drafted the manuscript. TS and FP critically revised the manuscript for important intellectual content. FP and RT supervised the study.

## Data Availability

The data that support the findings of this study are available on request from the corresponding author. The data are not publicly available due to privacy or ethical restrictions.
